# Synthesis of Janus plasmonic–magnetic, star–sphere nanoparticles, and their application in SERS detection

**DOI:** 10.1039/c6fd00012f

**Published:** 2016-03-02

**Authors:** Javier Reguera, Dorleta Jiménez de Aberasturi, Naomi Winckelmans, Judith Langer, Sara Bals, Luis M. Liz-Marzán

**Affiliations:** a CIC biomaGUNE , Paseo de Miramon 182 , 20009 Donostia-San Sebastián , Spain . Email: jreguera@cicbiomagune.es; b Ikerbasque , Basque Foundation for Science , 48013 Bilbao , Spain; c Biomedical Research Networking Center in Bioengineering Biomaterials and Nanomedicine , Ciber-BBN , Spain; d EMAT – University of Antwerp , Groenenborgerlaan 171 , B-2020 Antwerp , Belgium

## Abstract

Multicomponent nanoparticles are of particular interest due to a unique combination of properties at the nanoscale, which make them suitable for a wide variety of applications. Among them, Janus nanoparticles, presenting two distinct surface regions, can lead to specific interactions with interfaces, biomolecules, membranes *etc.* We report the synthesis of Janus nanoparticles comprising iron oxide nanospheres and gold nanostars, through two consecutive seed-mediated-growth steps. Electron tomography combining HAADF-STEM and EDX mapping has been performed to evaluate the spatial distribution of the two components of the nanoparticle, showing their clear separation in a Janus morphology. Additionally, SERS measurements assisted by magnetic separation were carried out to assess the application of combined plasmonic and magnetic properties for sensing.

## 1 Introduction

When the dimensions of a material decrease to the nanometric range, unique optical, electronic or magnetic properties may arise, making the resulting nanomaterial useful for a wide set of novel applications. Unlike the bulk material, such properties are dependent on the size and shape of the nanomaterial and can be easily combined through the use of multicomponent nanoparticles. This combination of properties can be exploited to achieve more complex tasks or give rise to new applications. A case of special interest is that of Janus nanoparticles, *i.e.* nanoparticles presenting two chemically different surface domains, which make them particularly interesting in applications where a spatial separation of functionalities is required.^1–3^ For example, the use of hydrophobic and hydrophilic functionalities generates “nanoamphiphiles” that produce strong adsorption at interfaces and emulsion stabilization,^4,5^ creating in some cases thermodynamically stable emulsions.^6,7^ Their use has also been proposed in heterogeneous catalysis,^8,9^ drug delivery,^10^ nanojet motors,^11^ antireflecting surfaces,^12^ or just as building blocks to form more complex structures such as supracrystals or molecular colloids.^13,14^ Despite the high interest in the use of Janus multicomponent nanoparticles, their applicability is still restricted by the limited current synthesis capabilities to obtain nanoparticles with different morphologies, shapes, and materials.

We present a new method that makes use of consecutive seed mediated growth steps to obtain complex nanoparticles with Janus morphology. The resulting nanoparticles comprise two different materials, iron oxide and gold, which present different properties. Iron oxide nanoparticles below a certain size exhibit superparamagnetism,^15^ which allows external manipulation by moderate magnetic fields, as well as hyperthermia through alternate magnetic fields.^16^ Iron oxide nanoparticles have also been used as contrast agents in magnetic resonance imaging (MRI).^17^ Gold nanoparticles, on the other hand, exhibit localized surface plasmon resonances (LSPR), which results in efficient absorption and scattering of light at specific wavelengths that can be tuned through the size and shape of the nanoparticles.^18^ This makes them attractive candidates as colorimetric sensors,^19^ nanoprobes for surface-enhanced Raman spectroscopy (SERS),^20^ labels for imaging,^21^ or targeting agents for photothermal treatment of cancer tissue.^22,23^ Among the numerous examples of gold nanoparticles, gold nanostars (NSs) are of interest because of their strong light absorption at the near-infrared (NIR) transparency window of biological tissue^24^ as well as the high electric field enhancements that can be induced at their tips,^25,26^ rendering them highly effective as SERS nanoprobes.^27^ Both gold and iron oxide nanoparticles can be easily functionalized, with thiols and amines in the case of gold, and catechols, silanes, diols carboxyls or amines in the case of iron oxide. They are also biocompatible materials so that they are often used in biomedical applications such as drug delivery, biolabelling, hyperthermia treatments, *etc.*

Several approaches have been reported in the literature to produce nanoparticles comprising gold and iron oxide parts, with different morphologies. These include: core–shell spherical nanoparticles,^28^ core–shell nanoflowers,^29^ core–shell nanostars,^30–32^ polymeric assemblies combining iron oxide nanoparticles with nanoparticles or nanostars,^33,34^ or porous silica containing iron oxide nanoparticles and gold nanorods.^35^ Janus dumbbell-like nanoparticles made of gold and iron oxide have been previously reported, exhibiting magnetic and plasmonic behavior,^36,37^ but the gold lobe is typically small (∼5–10 nm) and smaller than the iron oxide lobe (∼10–20 nm). This results in a weak LSPR band that in general overlaps partially with the scattering by the iron oxide part, thereby hindering applications where a strong plasmonic behavior is necessary.

Here we report the synthesis of Janus iron oxide–gold magnetic nanostars (JMNSs) with a strong plasmonic absorbance, using consecutive steps of seed-mediated-growth starting from gold nanoparticles through dumbbell-like nanoparticles, to JMNSs. UV-Vis spectroscopy and bright field transmission electron microscopy (TEM) were used to evaluate the optical properties and the morphology of the nanoparticles. The spatial distribution of gold and iron oxide in the nanoparticle shows a clear Janus character, as observed in 3D electron tomography combining high angle annular dark field scanning transmission electron microscopy (HAADF-STEM) and energy-dispersive X-ray spectroscopy (EDX) chemical mapping. Finally, as an example of the capabilities of these multifunctional nanoparticles, SERS sensing was performed to detect several dye molecules at low volumes and concentrations. Furthermore, thanks to the magnetic response of these nanoparticles, SERS measurements were performed in magnetically concentrated aggregates producing an effect of amplified SERS detection, which allows detection of much lower concentrations.

## 2 Results and discussion

**Fig. 1:**
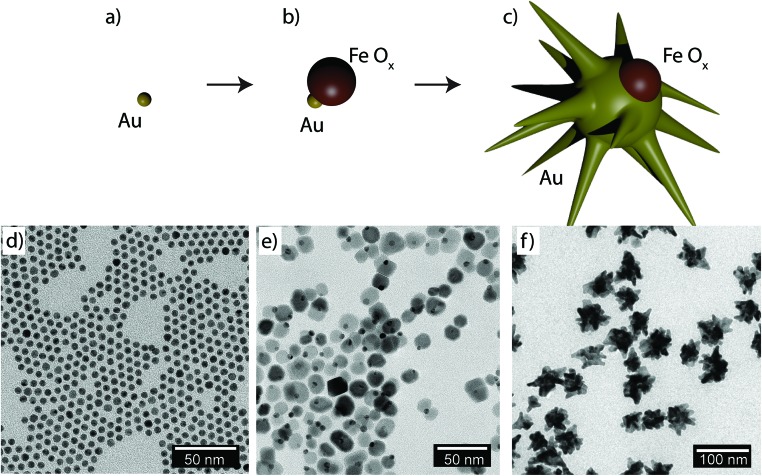
Scheme of the synthesis of Janus magnetic nanostars through consecutive seed-and-growth-steps. (a) Oleylamine synthesis of Au nanoparticles. (b) Growth of an iron oxide nanoparticle through the decomposition of Fe(CO_5_) and subsequent oxidation. (c) Growth of an Au nanostar coated with PVP using the NDs as seeds (d–f) TEM images of different nanoparticles corresponding to the scheme above: (d) Au NPs, (e) NDs, (f) JMNSs.

**Fig. 2:**
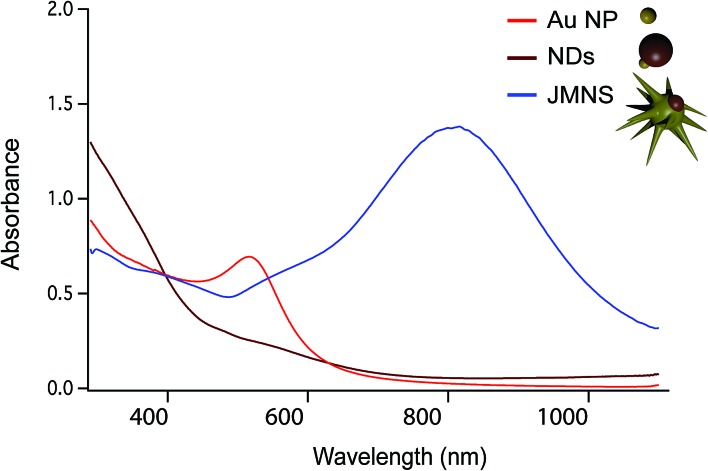
UV-Vis spectra comparing the absorbance of Au NPs (in red), NDs (in brown), and JMNSs (in blue).

Fig. 1a–c shows schematically the different steps performed to obtain Janus nanostars. Steps (a) and (b) were performed following the protocol described by Yu *et al.* for the synthesis of nanodumbbells (NDs).^36^ This synthesis can be performed in two different ways. The first method includes two separate steps where gold nanoparticles (Au NPs) are first synthesized with oleylamine and then used as seeds to grow the Fe part by thermal decomposition of Fe(CO)_5_. The second method, which has been used here, is a one-pot two-step method where HAuCl_4_ is injected into a solution at 120 °C containing oleylamine, oleic acid, hexadecanodiol, and Fe(CO)_5_. Au NPs immediately formed after injection; the temperature was then raised up to 300 °C to decompose Fe(CO)_5_, giving rise to Fe atoms that grow only on one facet of the Au seed. Yu *et al.* described the mechanism by which Fe grows on only one side of Au NPs.^36^ As Fe starts to nucleate on Au, the free electrons from Au compensate for the charge induced by the polarized plane at the interface. As the Au particle has only a limited source of electrons, this compensation makes all other facets of the Au nanoparticle electron deficient and unsuitable for multi-nucleation, resulting in the dumbbell structure. Once the synthesis of the Fe–Au dumbbells is completed, the nanoparticles are exposed to air, oxidizing Fe into a magnetic iron oxide, either as Fe_3_O_4_ or as γ-Fe_2_O_3_. We have gone one step further using these ND nanoparticles as seeds for a subsequent seed mediated growth step. With this aim, we performed a ligand exchange of the native capping agent by polymer ligands that allow dispersion in aqueous solution. In this case we used silane-poly(ethyleneglycol) and thiol-poly(*N*-isopropylacrylamide), which bind to the iron oxide and the gold surfaces, respectively. Modifying the polyvinylpyrrolidone (PVP)-assisted Au nanostar synthesis method,^38^ the growth of gold nanostars was performed by injection at room temperature of the dumbbell seeds in a pre-reduced DMF solution of HAuCl_4_. The formation of nanostars was observed by a color change from yellow to blue. Bright field TEM images of NDs and JMNSs are shown in Fig. 1e and f, compared with Au NPs synthesized without the iron oxide component and with sizes (*d* = 5.4 nm) similar to the Au part on the NDs (Fig. 1d). On those TEM images the increase in particle size and the morphological changes can be observed after the consecutive synthesis steps. Along with these morphological changes there is a concomitant change in the absorbance spectrum. Fig. 2 shows the UV-Vis spectra of the three kinds of nanoparticles, JMNSs, NDs and Au NPs. It can be observed that Au NPs feature an LSPR band centered at 520 nm, whereas for NDs the plasmon absorbance red-shifts to ∼560 nm, with a lower and broader absorption peak. When the nanostars are grown an intense LSPR band, typical for highly branched morphologies, is observed in the NIR (750–850 nm) which completely damps the iron oxide contribution. This absorbance at the NIR can be highly beneficial in biomedical applications as it is located at the first NIR absorbance window of living tissue and can be used to trigger externally a photothermal effect at the desired region of interest inside the human body.^24^

**Fig. 3:**
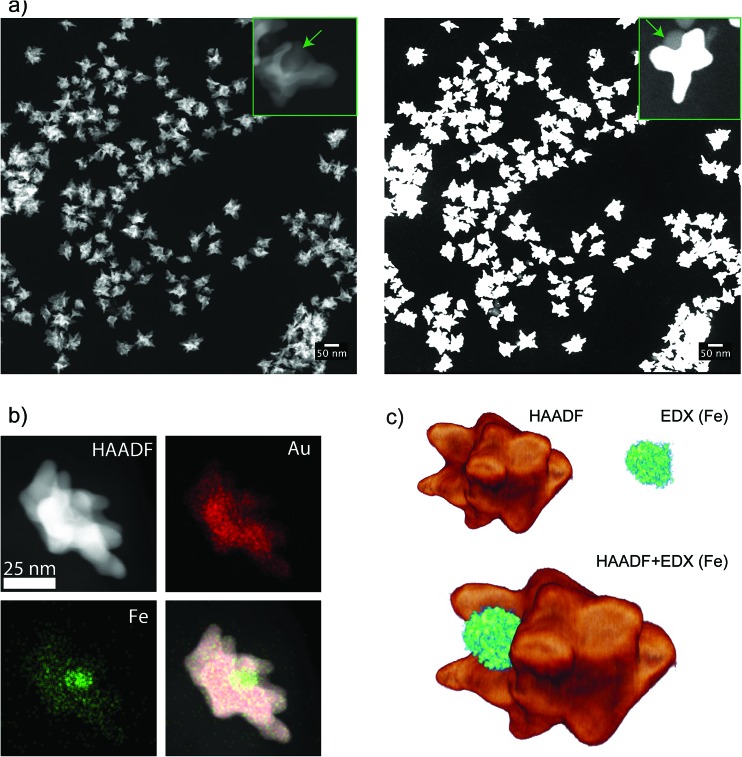
(a) HAADF-STEM image of JMNSs showing uniformity in size, the inset on the left shows a nanoparticle with a cavity corresponding to the iron oxide component. The image on the right shows the same area but saturating the detector, the inset shows a nanoparticle where the iron oxide part is exposing its surface in a clear Janus configuration. (b) Images of the same nanoparticle using HAADF-STEM, EDX mapping of gold atoms, EDX mapping of Fe atoms and a combination of the three. (c) Tomographic reconstruction of a nanoparticle combining HAADF-STEM tomography and EDX mapping of Fe, at different angles between –75° and 75° at 10° intervals.

Contrary to dumbbells (Fig. 1e), the Janus character of the nanostars (Fig. 1f) can be hardly appreciated in bright field TEM images due to the lower contrast of iron oxide as compared to Au and the larger nanostar tips. Thus, confirmation of the Janus character required the use of HAADF-STEM (Fig. 3a). In HAADF-STEM mode, the image intensity is determined by the average atomic number *Z* integrated over the thickness of the sample. Therefore, regions containing Au typically appear with a higher intensity than Fe-rich regions, as indicated in the insets of Fig. 3a. This figure suggests that practically all nanoparticles contain an iron oxide lobe and that this lobe could be exposed on one side of the nanostar. To confirm this observation and to investigate the 3D distribution of the elements, 3D EDX chemical mapping was performed. Therefore, a tilt series of 2D EDX maps was acquired over a tilt range of ±75°. An example of a 2D EDX map for Fe and Au is presented in Fig. 3b, together with an overlay of the maps with an HAADF-STEM image. From these images, a separation of the two components of the nanoparticles can already be distinguished. After using the tilt series of 2D EDX maps as an input for 3D reconstruction, the results presented in Fig. 3c are obtained. The 3D view allows a complete visualization of the nanoparticles, which indicates that the nanostar tips grow towards the opposite and lateral sides of the initial dumbbell nanoparticles, not covering the iron oxide and therefore leaving two different surfaces exposed for further selective functionalization.

**Fig. 4:**
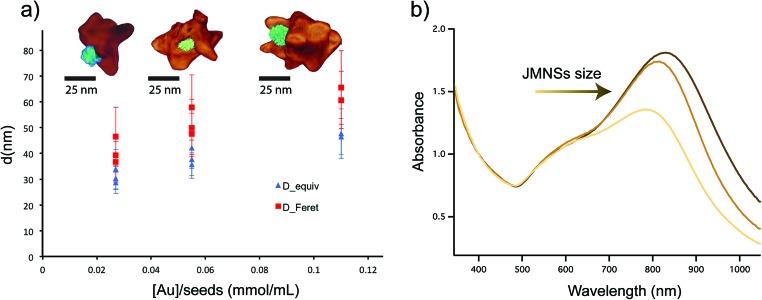
(a) Variation of JMNSs size depending on the synthesis conditions, *i.e.* varying the ratio between the amount of added ND seeds and HAuCl_4_ concentration. The size was evaluated as the average diameter by calculating the area from TEM images and assuming a spherical morphology (D_equiv., in blue) and secondly the Feret's maximum diameter (D_Feret, in red), which corresponds to the maximum tip-to-tip diameter. The 3D tomography images corresponding to each size show the presence of nanostar tips in all of them but an increase in the number of tips per nanoparticle. (b) UV-Vis spectra comparing the absorbance of the JMNSs with three different nanoparticle sizes shown in (a).

A similar analysis was performed on nanoparticles synthesized with different Au-to-seed ratios. Fig. 4a shows the total size of the nanoparticle measured as either average diameter or the maximum tip-to-tip diameter. As expected, an increase of the nanoparticle size is produced when less seeds per gold atom are added to the solution. The synthesis is fairly reproducible as can be seen for the different points corresponding to the same Au–Fe ratios. For intermediate values, two different concentrations of gold were used, indicating that the ratio between the amount of seeds and Au was the main parameter that defines the final particle size. Although the limit of nanoparticle sizes and ratios was not explored in this work, JMNPs were successfully synthesized between 30 and 50 nm. Elemental analysis by inductively coupled plasma mass spectrometry (ICP-MS), showed that the molar ratio between gold and iron varied from [Au]/[Fe] = 74 for the larger nanoparticles to [Au]/[Fe] = 13 for the smaller ones. Electron tomography was also performed on these nanoparticles to evaluate the morphological variation with nanoparticle size (Fig. 4a). The obtained 3D images invariably showed the Janus character of the nanoparticles, with gold branches growing in directions opposite to the iron oxide. Not much difference was observed in tip size and shape for big and small nanoparticles, but a clear increase was observed in the number of tips along with the size of the nanoparticle, *i.e.* bigger nanoparticles are more branched or spiky. Fig. 4b shows the spectra for the three different sizes, displaying the typical optical response of gold nanostars with a main LSPR mode corresponding to the tips around 700–800 nm and a secondary mode (shoulder around 600 nm) corresponding to the central core of the nanoparticles.^38,39^ Additionally, the decrease of the size of JMNSs produces a blue-shift of the band corresponding to the main LSPR mode while no big change is observed for the secondary mode. This change in the main mode could be attributed to the lower amount of tips per nanoparticle observed in the tomography images, and a possible variation in the thickness of those tips. These nanoparticles have high colloidal stability, as it has been seen for nanostars stabilized by PVP.^40^ In addition, different to the surfactant-free synthesis of nanostars, the PVP stabilization prevents reshaping of the nanostars.^40^ After 6 months the JMNSs were still dispersed in water and only a small blue-shift of ∼15 nm was observed in the UV-Vis absorption, indicative of a very small rounding of the nanostar tips over time.

**Fig. 5:**
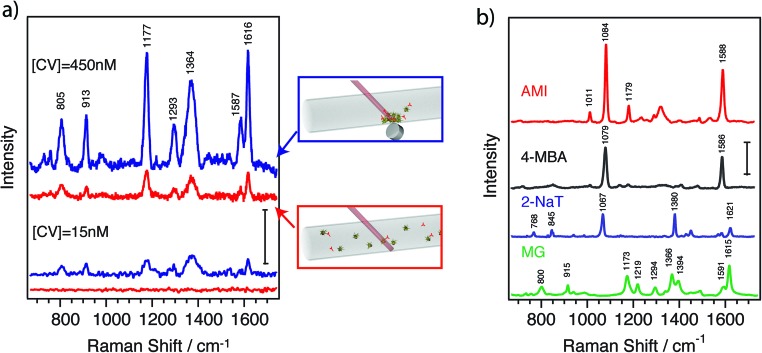
(a) SERS spectra of crystal violet [CV] containing JMNPs in solution (red) and after magnetic concentration (blue) for two different dye concentrations [CV] = 450 nM (upper spectra) and [CV] = 15 nM (lower spectra). Concentration of JMNSs is [Au^0^] = 0.2 and 0.1 mM respectively. The spectra were shifted vertically for clarity with the scale bar corresponding to an intensity of 200 counts. (b) SERS spectra of different molecules after a magnetic aggregation. AMI: acetoamidothiophenol, 4-MBA: 4-mercaptobenzoic acid, 2-NaT: 2-naphthalenethiol, MG: malachite green. Analyte concentration 4.5 μM, [Au^0^] = 0.2 mM. AMI, 4-MBA and 2-NaT were measured with 785 nm excitation whereas MG was measured with a 633 nm excitation wavelength. The intensity scale bar corresponds to 500 counts.

To assess the plasmonic and magnetic properties of these nanoparticles in a direct application, SERS was performed for detection of selected molecules. Fig. 5 shows the SERS spectra of an analyte solution containing the model analyte crystal violet (CV) with a concentration of 450 nM (red, upper spectrum). The spectrum clearly shows the characteristic peaks of CV at 1616, 1597, 1293 (assigned as C–C stretching), 1377 (*N*-phenyl-stretching), 1175 (C–H in-plane deformation), 915 and 805 cm^–1^ (C–H out-of-plane deformation) with no external interference.^41^ A second spectrum was recorded on the same sample after applying a magnetic field (Fig. 5a, blue line). For this purpose, a small magnet was placed close to the solution for 30 minutes. The nanoparticles magnetically aggregated close to the magnet, thereby increasing the concentration where the laser spot of the Raman spectrometer was focused. Remarkably, we registered SERS signals with an enhancement factor of ∼5 times with respect to the signal before magnetic concentration. The measurements produce reproducible results for the magnetically aggregated samples down to CV concentrations of 15 nM (Fig. 5a). At such low concentrations no signal can be observed when the measurement is performed in solution without applying a magnetic field (red spectrum). Below 15 nM the spectra are less reproducible due to the low signal-to-noise ratio. The observed increase in signal is most likely due to adsorption of the analytes on the surface of the nanoparticles. When they are in solution, the diffusion time from the analyte position to the surface of a nanoparticle can be very short, making the “capture” of the analyte quite effective. Upon application of a magnetic field, the nanoparticles drag the analytes to a higher concentration area, thereby increasing the measured SERS signal. It should be noted that an optimization of the amount of nanoparticles would be necessary to decrease the detection limit, as too many nanoparticles lead to an increase of the background signal and few nanoparticles decrease the surface available for capturing the analyte.

We additionally carried out SERS measurements with several molecules that have affinity for the gold surface, including molecules containing amino and thiol groups. Fig. 5b shows SERS spectra from solutions containing different molecules at 4.5 μM after magnetic aggregation. At the same concentration, JMNSs yielded clear SERS spectra for all the molecules containing thiols or amines tested here. Furthermore, SERS spectra do not show any meaningful signal from PVP, which was used to stabilize the nanoparticles, or from any other reagent used in the synthesis, resulting in clearly assignable SERS spectra for all the analytes.^34,39,42–46^ On the other hand, PVP is a weakly adsorbed polymer, so it can be readily replaced by other molecules or polymers that present a higher affinity for the desired analytes, making the nanoparticles more specific and effective for their detection.

## 3 Experimental

### 3.1 Synthesis

#### 3.1.1 Synthesis of dumbbell-like nanoparticles

A solution in 1-octadecane (40 mL) was prepared containing oleic acid (6 mmol), oleylamine (6 mmol) and 1,2-hexadecanodiol (10 mmol) and stirred for 20 min at 120 °C under N_2_. Fe(CO)_5_ (0.3 mL) was then injected and after 3 min a solution containing HAuCl_4_·3H_2_O (0.1 mmol) dissolved in a mixture of oleylamine (0.5 mL) and 1-octadecane (5 mL) was injected and heated up to 300 °C at approximately 1 °C min^–1^. The solution was left to react for 45 min. After cooling down, the dispersion was exposed to air for 30 min to cause Fe oxidation.

To purify the nanoparticles, 50 mL of isopropanol was added and the solution centrifuged at 4500 RCF for 30 min. The nanoparticles were cleaned two more times after the redispersion with hexane and aggregation with isopropanol. Finally oleylamine (100 μL) was added to store the nanoparticles for long periods of time. The nanoparticles were cleaned again right before functionalization. The final nanoparticle sizes were 5.3 ± 0.8 nm for the Au part and 16.2 ± 2.8 nm for the iron oxide part.

#### 3.1.2 Ligand exchange

Silane-poly(ethylene glycol) (MW = 5 kg mol^–1^) was purchased from Creative PEGWorks. HS-poly(*N*-isopropylacrylamide) (MW = 12 kg mol^–1^) was synthesized by RAFT polymerization using ethyl 4-(hydoxymethyl)benzyl carbonotrithioate as RAFT agent and 4,4′-azobis 4(-cyanopentanoic acid) as initiator. 11 mg of nanoparticles was incubated in toluene (20 mL) together with HS-PNIPAM (60 mg, MW 12 kg mol^–1^), and PEG-silane (110 mg). Acetic acid (3 μL) was added to induce polycondensation of the silane. The solution was sonicated for 10 min and then left shaking during 2 days. The nanoparticles were purified by transferring into water by adding a small amount of hexane to the solution. The nanoparticles were further purified in water several times using a centrifugation filter (pore size 50 kDa) and finally redispersed in water (10 mL).

#### 3.1.3 Synthesis of Janus nanostars (JMNSs)

A solution of HAuCl_4_·3H_2_O (54.6 μL, 50 mM) was added to a solution containing polyvinylpyrrolidone (1 g, MW = 10 kg mol^–1^) dissolved in DMF (10 mL). The solution was left stirring for 20 min to allow gold salt reduction. The dumbbell nanoparticle solution (25 μL, except those in Fig. 4 described below) was then added and the reaction was left reacting for 1 h, showing a colour change into blue. The concentration of dumbbells and JMNSs was estimated by ICP-MS (see below). The nanoparticles were purified in several centrifugation cycles: the first one at 3420 g for 1 h, 2 more at 2870 g for 1 h and 2 more at 2370 g RCF for 15 min. They were finally redispersed in Milli-Q water.

##### JMNSs of different sizes

Different nanoparticles were obtained by changing the ratio between Au and the amount of ND seeds. JMNS1 are described above. JMNS2 and JMNS3 have the same [Au] : seed ratio and showed similar sizes.

JMNS1: 25 μL of seeds, 54.6 μL of Au salt (50 mM) in 10 mL of DMF.

JMNS2: 50 μL of seeds, 54.6 μL of Au salt (50 mM) in 10 mL of DMF.

JMNS3: 25 μL of seeds, 27.3 μL of Au salt (50 mM) in 10 mL of DMF.

JMNS4: 50 μL of seeds, 27.3 μL of Au salt (50 mM) in 10 mL of DMF.

#### 3.1.4 Synthesis of Au nanoparticles

To compare the plasmonic behavior of the dumbbells, Au nanoparticles were synthesized with oleylamine to have a similar size. HAuCl_4_·3H_2_O (0.5 g) was dissolved in a mixture of oleylamine (40 mL) and 1,2,3,4-tetrahydronaphthalene (40 mL) at room temperature under gentle N_2_ bubbling. Oleylamine (15 mL), 1,2,3,4-tetrahydronaphthalene (15 mL) and a *tert*-butylamineborane complex (2.5 mol) were dissolved and added to the initial solution. The reaction was allowed to proceed for 1 h. Purification was performed by adding isopropanol (320 mL) and centrifuging. Two additional centrifugation steps were performed by redispersing the nanoparticles and precipitating them with isopropanol. Finally, the nanoparticles were dispersed in hexane (20 mL), and oleylamine (200 μL) was added prior to storage. The final nanoparticle size was measured by TEM assuming a spherical shape. Diameter = 5.4 ± 0.4 nm.

### 3.2 Characterization methods

#### 3.2.1 UV-Vis spectroscopy

Optical extinction spectra were recorded using an Agilent 8453 UV/Vis diode-array spectrophotometer. The resulting spectra were normalized at 400 nm. At this wavelength the absorbance by gold nanoparticles has been shown to be nearly independent of particle size.^47^

#### 3.2.2 ICP-MS

With the aim of obtaining the atomic ratio between metals (Fe and Au) in the JMNs, analysis by inductively coupled plasma mass spectrometry (ICP-MS) (Agilent 7500ce) was performed. An internal calibration was performed using yttrium at a 500 ppb concentration. Measurements were carried out for three different nanoparticle colloids: nanodumbbell seeds (NDs), big Janus magnetic nanostars JMNSs (JMNS1, nanoparticles shown in Fig. 3) and small Janus magnetic nanostars (JMNS4, small nanoparticles shown in Fig. 4). All particles were digested with 30 vol% aqua regia before the ICP analysis.

— NDs: the obtained results yielded 0.29 mg mL^–1^ of Fe and 0.14 mg mL^–1^ of Au.

— JMNS1: 18.24 mg mL^–1^ of Au and 0.07 mg mL^–1^ of Fe.

— JMNS4: 4.03 mg mL^–1^ of Au to 0.09 mg mL^–1^ of Fe.

#### 3.2.3 Bright field transmission electron microscopy (TEM)

TEM bright field images were acquired in a JEOL JEM-1400PLUS instrument operating at 120 kV. Sizes of nanoparticles were determined from bright field images with more than 200 nanoparticles. The images were analyzed with the Image J software package. Due to the difficulty of measuring these morphologically complex nanoparticles two different diameters are provided, allowing a comparison between different nanoparticles. An equivalent diameter was obtained by calculating the projected area and assuming a spherical morphology. A second diameter was obtained by calculating the maximum Feret's diameter, *i.e.* the length between two parallel lines that surround the nanoparticle. This diameter corresponds to the maximum tip-to-tip distance of the 2D projected nanoparticle and could thus be slightly underestimated due to the 3D morphology of the nanoparticles.

#### 3.2.4 Electron tomography

HAADF-STEM images, EDX mapping and electron tomography tilt series were acquired using a model 2020 Fischione Instruments tomography holder and a FEI Tecnai Osiris electron microscope operated at 200 kV. A camera length of 87 mm was used. The tilt series of HAADF-STEM and EDX are recorded at angles ranging from +75° to –75° with a 10° increment. For the reconstruction of the series we used the SIRT algorithm, as implemented in the ASTRA toolbox.^48^

#### 3.2.5 Surface enhanced Raman spectroscopy

SERS measurements were performed by means of a confocal Raman microscope (Renishaw inVia) equipped with a 1024 × 512 CCD Peltier-cooled detector using either 633 nm excitation laser with maximal output of 17 mW combined with a 1800 L mm^–1^ diffraction grating or 785 nm excitation laser with maximum output of 270 mW, combined with a 1200 L mm^–1^ diffraction grating. 20 μL of the magnetic Janus particles in aqueous solution ([Au^0^] = 0.2 mM) were filled in cut Pasteur glass pipettes (1 mm diameter) and mounted on a home-built holder. For measurements in aggregated samples, the edge of a neodymium magnetic disc (1.42 T) was placed below the glass tube and SERS spectra were measured after 1 h. For measurements in non-aggregated samples the same holder without the magnet was used. All SERS spectra were collected using a 10× objective (numerical aperture NA = 0.35) with an integration time of 2 s and a laser power of 5 mW for 633 nm (for CV and MG) accumulating 50 cycles and 16 mW for 785 nm (for AMI, 4-MBA and 2-NaT) accumulating 100 cycles. All spectra presented here are averaged before background correction using the Wire 3.4 software.

## 4 Conclusions

We have demonstrated in this work that consecutive seed-mediated-growth steps lead to production of complex nanostructures that can be used in sophisticated applications by combining different properties. In this case, nanoparticles were synthesized in three steps, where two seed-mediated-growth steps lead from gold nanoparticles to Janus or dumbbell like nanoparticles, and Janus iron oxide–gold nanosphere–nanostar nanoparticles. These nanoparticles feature superparamagnetic and plasmonic properties and asymmetric activity due to their Janus character, which are of high potential for diverse applications ranging from interface stabilization of oil–water emulsions with magneto-plasmonic properties to biomedical applications. 3D imaging by tomography based on EDX chemical mapping proved to be an effective method to visualize complex morphologies. The 3D images of these nanoparticles revealed that the growth of the gold tips avoided the surface of the iron oxide nanoparticle, producing Janus nanoparticles with two clearly differentiated surfaces. Moreover, an increase in the size of the gold nanoparticle together with an increase on the number of star tips was produced with the increase of HAuCl_4_ used on the synthesis of the Janus magnetic nanostars. We finally proved that the plasmonic and magnetic properties of these nanoparticles can be effectively used in a direct application. The particles were used as nanoprobes for SERS measurements in solution thanks to their effective plasmonic properties, without any further surface modification. Furthermore, taking advantage of their magnetic properties, they were magnetically concentrated (magnetic extraction) to produce an additional enhancement of the signal that allowed detection down to 15 nM. Further work would be necessary to functionalize the nanoparticles allowing more specific and effective Raman enhancement of relevant biomolecules. We propose that the Janus character will open new possibilities in applications that require strong interaction with interfaces or in cases where specific functional groups need to be spatially separated, in a similar way as proteins and molecules present different patchy surfaces to achieve their programmed biological functions.
